# Supplementary material for: Survival Improvement over Time of 960 s-AML Patients Included in 13 EORTC-GIMEMA-HOVON Trials
Source: Cancers (Basel). 2020 Nov 11;12(11):3334. doi: 10.3390/cancers12113334 (PMC7697114; doi:10.3390/cancers12113334)
Supplement: Supplementary file 1 [file cancers-12-03334-s001.pdf]

Supplemental Materials:

Survival Improvement over Time of 960 s-AML Patients Included in 13 EORTC-GIMEMA-HOVON Trials

Safaa M. Ramadan, Stefan Suciu, Marian J. P. L. Stevens-Kroef, Roelof Willemze, Sergio Amadori, Theo de Witte, Bob Löwenberg, Petra Muus, Boris Labar, Liv Meert, Gaetan de Schaetzen, Giovanna Meloni, Giuseppe Leone, Marco Vignetti, Jean-Pierre Marie, Michael Lübbert and Frédéric Baron

Table S1. Prospective trials which contributed patients to the current study.

Protocol# (reference of the publication)	Title	Study details
06012 [1]	Gemtuzumab Ozogamycin (GO) combined with standard intensive chemotherapy versus standard intensive chemotherapy alone for induction/consolidation in patients 61 - 75 years old with previously untreated AML: a randomized phase III trial (AML-17) of the EORTC - LG and the GIMEMA-ALWP	https://www.eortc.org/research_field/clinical-detail/06012/
06013 [2]	Idarubicin and Ara-C in combination with Gemtuzumab-Ozogamycin (IAGO) for untreated patients, with or without an HLA identical sibling, with high risk MDS or AML developing after a preceding period with MDS during 6 months duration: a phase II study.	https://www.eortc.org/research_field/clinical-detail/06013/
06862 [3]	A controlled phase III study on the value of intensive remission induction and remission maintenance chemotherapy in elderly patients with acute myelogenous leukemia (AML-9)	https://www.eortc.org/research_field/clinical-detail/06862/
06863 [4,5]	A randomized phase III study of autologous bone marrow transplantation versus intensive consolidation during first complete remission in acute myelogenous leukemia (AML-8A)	https://www.eortc.org/research_field/clinical-detail/06863/
06864 [6]	A randomized phase III study of intensive consolidation with high dose cytosine arabinoside in acute myelogenous leukemia (AML-8B)	https://www.eortc.org/research_field/clinical-detail/06864/
06888 [7]	A pilot (phase II) study of intensive remission induction chemotherapy for bad prognosis myelodysplastic syndromes (MDS) and acute myelogenous leukemia secondary (sAML) tp MDS of more than 6 months duration in patients with age younger than 60 years	https://www.eortc.org/research_field/clinical-detail/06888/
06892 [8]	A randomized phase III study on the value of GM-CSF administration as an adjunct to intensive remission induction chemotherapy in elderly patients with acute myelogenous leukemia	https://www.eortc.org/research_field/clinical-detail/06892/
06921 [9]	A pilot phase II study of an intensified remission induction chemotherapy followed by an autologous or allogeneic bone marrow transplantation for bad prognosis myelodysplastic syndromes (MDS) and acute myelogenous leukemia secondary (sAML) to MDS of more than six months duration in patients with age younger than 60 years	https://www.eortc.org/research_field/clinical-detail/06921/
06931 [10]	Randomized phase III study of induction (ICE vs MICE vs DCE) and intensive consolidation (IDIA vs NOVIA vs DDIA) followed by allogeneic bone marrow transplantation or a randomized autologous BMT vs	https://www.eortc.org/research_field/clinical-detail/06931/

	autologous peripheral stem cell transplantation in acute myelogenous leukemia	
06954 [11]	Randomized phase III study to evaluate the value of rHuG-CSF in induction and of an oral schedule as consolidation treatment in elderly patients with acute myelogenous leukemia (AML-13 protocol) (Jointly with the GIMEMA)	https://www.eortc.org/research_field/clinical-detail/06954/
06961 [12]	A randomized phase III study for autologous peripheral blood stem cell transplantation (PSCT) vs a second intensive consolidation course after a common induction and consolidation course in patients with bad prognosis MDS and sAML to MDS of more than 6 months duration.	https://www.eortc.org/research_field/clinical-detail/06961/
06991 (AML-12) [13]	The value of high dose versus standard dose Ara-c during induction and IL-2 after intensive consolidation/autologous stem cell transplantation in patients (age 15-60 yrs) with acute myelogenous leukemia. A randomized phase III trial of the EORTC and GIMEMA Leukemia Cooperative Groups (AML-12).	https://www.eortc.org/research_field/clinical-detail/06991/
06993 [14]	Gemtuzumab Ozogamicin (CMA-676) followed or not by intensive chemotherapy as initial treatment for elderly patients with acute myeloid leukemia: an EORTC-LG pilot phase II study.	https://www.eortc.org/research_field/clinical-detail/06993/

Table S2. Baseline characteristics, treatment period and intensity in patients with s-AML following MDS (group A), other malignancies (group B) or non-malignant conditions (Group C), being 16 to 85 years old at the start of treatment.

	**Group A MDS (N=508)**	**Group B Other malignancies (N = 361)[a]**	**Group C Non-malignant conditions (N = 91)**	**Total (n = 960)**
**Year of inclusion, n (%)**				
< 1990	48 (9.4)	44 (12.2)	15 (16.5)	107 (11.1)
1990-2000	247 (48.6)	182 (50.4)	58 (63.7)	487 (50.7)
2000-	213 (41.9)	135 (37.4)	18 (19.8)	366 (38.1)
**Sex, n (%)**				
Male	304 (59.8)	159 (44.0)	52 (57.1)	515 (53.6)
Female	204 (40.2)	202 (56.0)	39 (42.9)	445 (46.4)
**Age (years)**				
Median (range)	64.0 (20.0 - 85.0)	60.0 (16.0 - 85.0)	61.0 (18.0 - 79.0)	63.0 (16.0 - 85.0)
16-45, n (%)	48 (9.4)	79 (21.9)	23 (25.3)	150 (15.6)
46-60, n (%)	133 (26.2)	107 (29.6)	22 (24.2)	262 (27.3)
61-69, n (%)	191 (37.6)	115 (31.9)	25 (27.5)	331 (34.5)
70-85, n (%)	136 (26.8)	60 (16.6)	21 (23.1)	217 (22.6)
**WHO PS, n (%)**				
0	199 (39.2)	123 (34.1)	28 (30.8)	350 (36.5)
1	232 (45.7)	168 (46.5)	44 (48.4)	444 (46.3)
2	68 (13.4)	58 (16.1)	18 (19.8)	144 (15.0)
3	9 (1.8)	10 (2.8)	1 (1.1)	20 (2.1)
**WBC × 10^9/L**				
Median (range)	4.6 (0.3 - 408.3)	8.9 (0.4 - 296.7)	3.3 (0.9 - 176.0)	5.3 (0.3 - 408.3)
< 25, n (%)	390 (76.8)	239 (66.2)	75 (82.4)	704 (73.3)
>= 25, n (%)	118 (23.2)	121 (33.5)	16 (17.6)	255 (26.6)
**Cytogenetic risk, n (%)**				
Good	2 (0.4)	24 (6.6)	1 (1.1)	27 (2.8)
Intermediate	238 (46.9)	143 (39.6)	37 (40.7)	418 (43.5)
Poor	86 (16.9)	53 (14.7)	16 (17.6)	155 (16.1)

Unknown	182 (35.8)	141 (39.1)	37 (40.7)	360 (37.5)
**Monosomal karyotype, # of pts (%)**				
No (MK-)	294 (57.9)	187 (51.8)	47 (51.6)	528 (55.0)
Yes (MK+)	30 (5.9)	30 (8.3)	7 (7.7)	67 (7.0)
Unknown	184 (36.2)	144 (39.9)	37 (40.7)	365 (38.0)
**Prior MDS**				
No	0 (0)	326 (90.3)	53 (58.2)	379 (39.5)
Yes	508 (100)	35 (9.7)	38 (41.8)	581 (60.5)
**Prior malignancy, n (%)**				
**No**	**508**	**0**	**91**	
**Hematological malignancy**	NA	**41 (11.4)**	NA	
NHL	NA	17 (4.7)	NA	
CMML	NA	11 (3.0)	NA	
Other	NA	13 (3.6)	NA	
**Solid malignancy**	NA	**232 (64.3)**	NA	
Breast	NA	87 (24.1)	NA	
Gynecologic	NA	28 (7.8)	NA	
Digestive - gastrointestinal	NA	23 (6.4)	NA	
Genitourinary	NA	34 (9.4)	NA	
Head and neck	NA	28 (7.8)	NA	
CNS	NA	5 (1.4)	NA	
Musculoskeletal	NA	4 (1.1)	NA	
Respiratory - thoracic	NA	7 (1.9)	NA	
Skin	NA	16 (4.4)	NA	
Unknown prior malignancy	NA	129 (35.7)	NA	
**Prior non-malignant disease**	NA	NA	**36 (39.6)**	
Autoimmune disease	NA	NA	5 (13.9)	
Anemia	NA	NA	7 (19.4)	
Thalassemia	NA	NA	4 (11.1)	
Cytopenia	NA	NA	5 (13.9)	
BM hypoplasia	NA	NA	2 (5.6)	
Other / Missing	NA	NA	13 (19.4)	
**Toxic exposure**	NA	NA	**55 (60.4)**	
Therapeutical	NA	NA	16	
Occupational	NA	NA	5	
Other / missing	NA	NA	34	

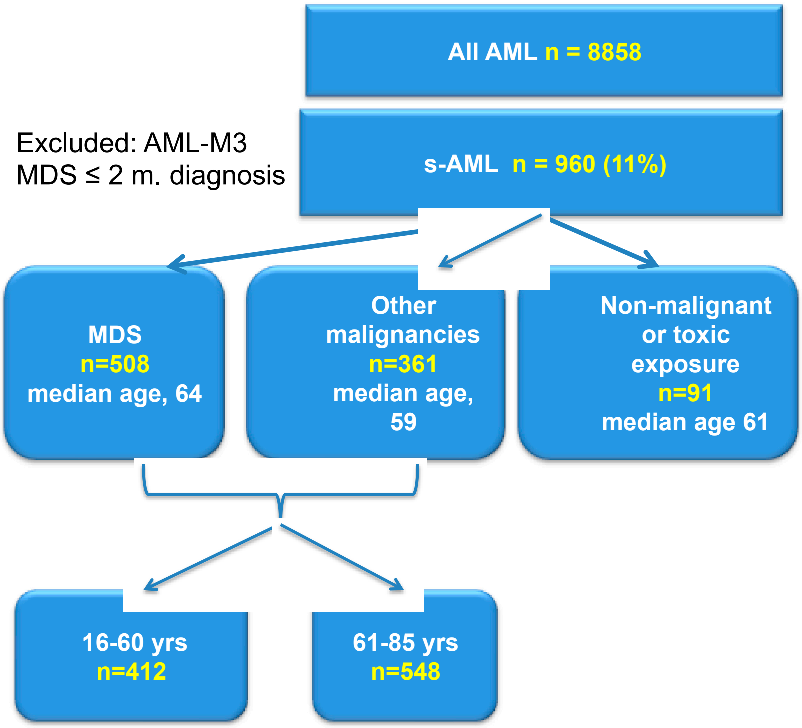

Figure S1. Flowshart of patients included in the sAML database.

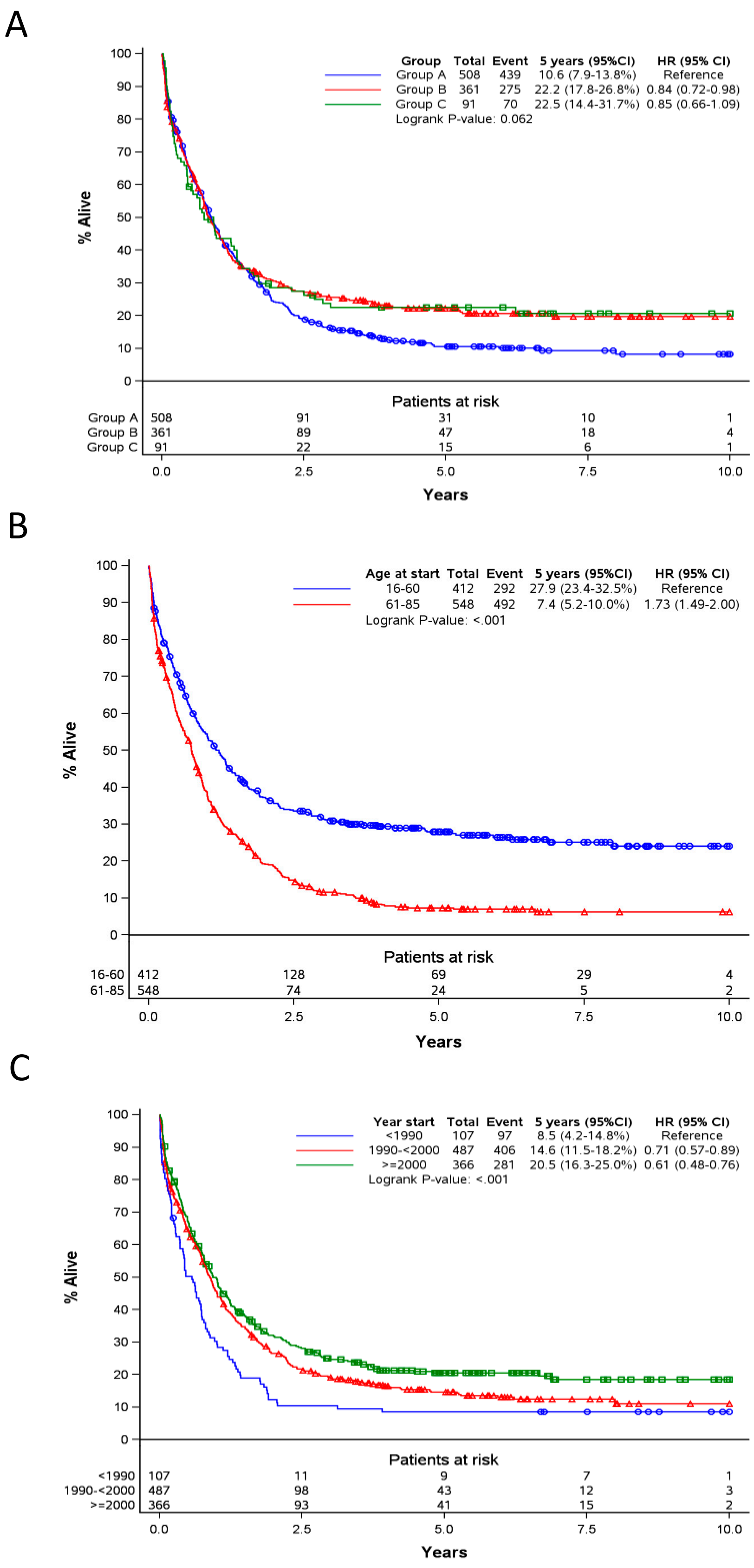

Figure S2. OS according to the cause of s AML (A), patients' age at diagnosis (B) and year of inclusion (C).

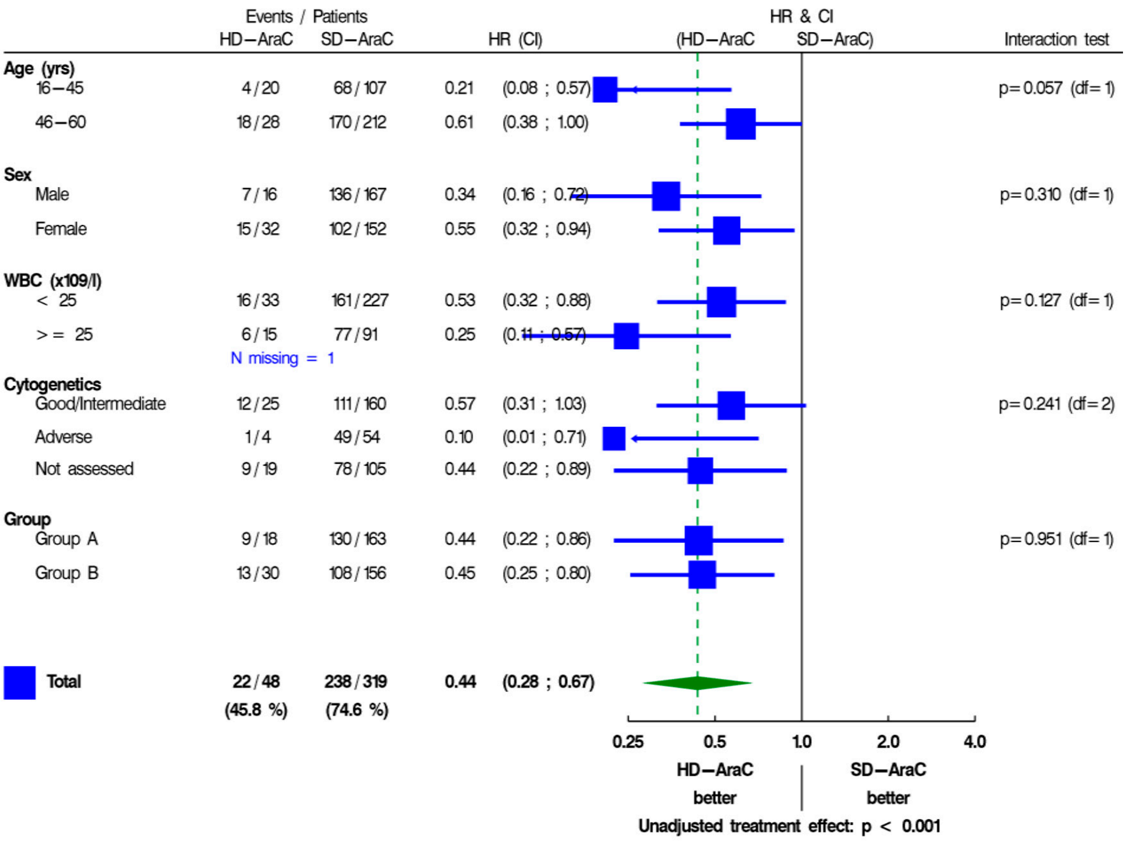

Figure S3. Forest plot of the impact of HD-AraC on OS in younger patients from group A or B.

References

1. Amadori S, Suciu S, Stasi R, et al. Sequential combination of gemtuzumab ozogamicin and standard chemotherapy in older patients with newly diagnosed acute myeloid leukemia: results of a randomized phase III trial by the EORTC and GIMEMA consortium (AML-17). *Journal of clinical oncology: official journal of the American Society of Clinical Oncology.* 2013;31(35):4424-4430.
2. de Witte T, Suciu S, Meert L, et al. Idarubicin and cytarabine in combination with gemtuzumab ozogamicin (IAGO) for untreated patients with high-risk MDS or AML evolved from MDS: a phase II study from the EORTC and GIMEMA Leukemia Groups (protocol 06013). *Ann. Hematol.* 2015;94(12):1981-1989.
3. Lowenberg B, Suciu S, Archimbaud, E., et al. Mitoxantrone versus daunorubicin in induction-consolidation chemotherapy--the value of low-dose cytarabine for maintenance of remission, and an assessment of prognostic factors in acute myeloid leukemia in the elderly: final report. European Organization for the Research and Treatment of Cancer and the Dutch-Belgian Hemato-Oncology Cooperative Hovon Group. *Journal of Clinical Oncology.* 1998;16(3):872-881.
4. Zittoun RA, Mandelli, F., Willemze, R., et al. Autologous or allogeneic bone marrow transplantation compared with intensive chemotherapy in acute myelogenous leukemia. *New England Journal of Medicine.* 1995;332:217-223.
5. Baron F, Efficace F, Cannella, L., et al. Long-term follow-up of a trial comparing post-remission treatment with autologous or allogeneic bone marrow transplantation or intensive chemotherapy in younger acute myeloid leukemia patients. *Haematologica.* 2019.
6. Hengeveld M, Suciu S, Karrasch M, et al. Intensive consolidation therapy compared with standard consolidation and maintenance therapy for adults with acute myeloid leukaemia aged between 46 and 60 years: final results of the randomized phase III study (AML 8B) of the European Organization for Research and Treatment of Cancer (EORTC) and the Gruppo Italiano Malattie Ematologiche Maligne dell'Adulto (GIMEMA) Leukemia Cooperative Groups. *Ann. Hematol.* 2012;91(6):825-835.
7. de Witte T, Suciu S, Peetermans, M., et al. Intensive chemotherapy for poor prognosis myelodysplasia (MDS) and secondary acute myeloid leukemia (sAML) following MDS of more than 6 months duration. A pilot study by the Leukemia Cooperative Group of the European Organisation for Research and Treatment in Cancer (EORTC-LCG). *Leukemia* 1995;9(11):1805-1811.

8. Amadori S, Suciu S, Jehn U, et al. Use of glycosylated recombinant human G-CSF (lenograstim) during and/or after induction chemotherapy in patients 61 years of age and older with acute myeloid leukemia: final results of AML-13, a randomized phase-3 study. *Blood.* 2005;106(1):27-34.
9. de Witte T, Suciu S, Verhoef G, et al. Intensive chemotherapy followed by allogeneic or autologous stem cell transplantation for patients with myelodysplastic syndromes (MDSs) and acute myeloid leukemia following MDS. *Blood.* 2001;98(8):2326-2331.
10. Mandelli F, Vignetti M, Suciu S, et al. Daunorubicin versus mitoxantrone versus idarubicin as induction and consolidation chemotherapy for adults with acute myeloid leukemia: the EORTC and GIMEMA Groups Study AML-10. *Journal of clinical oncology: official journal of the American Society of Clinical Oncology.* 2009;27(32):5397-5403.
11. Jehn U, Suciu S, Thomas X, et al. Non-infusional vs intravenous consolidation chemotherapy in elderly patients with acute myeloid leukemia: final results of the EORTC-GIMEMA AML-13 randomized phase III trial. *Leukemia.* 2006;20(10):1723-1730.
12. de Witte T, Hagemeijer A, Suciu, S., et al. Value of allogeneic versus autologous stem cell transplantation and chemotherapy in patients with myelodysplastic syndromes and secondary acute myeloid leukemia. Final results of a prospective randomized European Intergroup Trial. *Haematologica.* 2010;95(10):1754-1761.
13. Willemze R, Suciu S, Meloni G, et al. High-Dose Cytarabine in Induction Treatment Improves the Outcome of Adult Patients Younger Than Age 46 Years With Acute Myeloid Leukemia: Results of the EORTC-GIMEMA AML-12 Trial. *Journal of clinical oncology: official journal of the American Society of Clinical Oncology.* 2014;32(3):219-228.
14. Amadori S, Suciu S, Willemze R, et al. Sequential administration of gemtuzumab ozogamicin and conventional chemotherapy as first line therapy in elderly patients with acute myeloid leukemia: a phase II study (AML-15) of the EORTC and GIMEMA leukemia groups. *Haematologica.* 2004;89(8):950-956.

© 2020 by the authors. Licensee MDPI, Basel, Switzerland. This article is an open access article distributed under the terms and conditions of the Creative Commons Attribution (CC BY) license (http://creativecommons.org/licenses/by/4.0/).
